# Acute Renal Failure and Nephrotic Syndrome Secondary to Collapsing Glomerulopathy Associated With Hepatitis C

**DOI:** 10.7759/cureus.23175

**Published:** 2022-03-15

**Authors:** Brandon Wiggins, Smit Deliwala, Fady Banno, Kyle Knight, Mark Minaudo

**Affiliations:** 1 Internal Medicine, Ascension Health, Grand Blanc, USA; 2 Internal Medicine, Hurley Medical Center, Flint, USA; 3 Gastroenterology and Hepatology, Ascension Health, Grand Blanc, USA

**Keywords:** non-oliguric renal failure, hepatitis c management, collapsing glomerulonephritis, nephrotic, hepatitis c (hcv) infection

## Abstract

Collapsing glomerulopathy (CG) is a rare variant of focal segmental glomerulosclerosis (FSGS) that commonly presents as nephrotic syndrome in patients. CG is almost always associated with human immunodeficiency virus (HIV) infection but is rarely from other infectious sources such as parvovirus, Epstein-Barr virus, cytomegalovirus, and SARS-CoV-2. CG has also been reported to be related to other etiologies such as genetic disorders, lupus, malignancy, and post-renal transplant but is exceedingly rare when related to hepatitis C virus (HCV). In this report, we describe the case of a patient presenting with nephrotic syndrome secondary to CG caused by newly diagnosed HCV.

## Introduction

Hepatitis C virus (HCV) infection is a systemic disorder that has a global prevalence of 2.5% (177.5 million HCV-infected adults) [1]. It is often associated with several extrahepatic manifestations, including glomerular disease [2,3]. The most common HCV-associated glomerulopathy is membranoproliferative glomerulonephritis (MPGN) associated with mixed cryoglobulinemia followed by MPGN without cryoglobulinemia and membranous nephropathy [4]. Various histological types of renal diseases have been reported in association with HCV infection, including MPGN, membranous nephropathy, focal segmental glomerulosclerosis (FSGS), immunoglobulin A nephropathy, renal thrombotic microangiopathy, interstitial nephritis, and immunotactoid glomerulopathy [4-6].

Collapsing glomerulopathy (CG) is a rare variant of FSGS that represents approximately 4.7% of biopsies with FSGS [7]. In CG, cytokines, typically interferons, upregulate the expression of the apolipoprotein L1 (*APOL1*) gene in glomerular epithelial cells. Upregulation of mutant *APOL1* gene causes direct injury to these cells and leads to a collapsing pattern of glomerular capillaries, hypertrophic podocytes, and cystic dilation of tubules on histology [8]. CG is almost always related to human immunodeficiency virus (HIV) infection and histologically defines HIV-associated nephropathy (HIVAN) [9]. However, the variant may also be associated with other infections, such as parvovirus, Epstein-Barr virus, cytomegalovirus, and SARS-CoV-2, although this is rare [10].

CG has been described in the literature in the setting of renal transplant induced by medication, genetic disorders, idiopathic causes, and viral infections. It usually presents with nephrotic syndrome and renal insufficiency [11]. CG is well documented in the literature in association with HIV; however, to our knowledge, it has rarely been described in HCV infection [12,13].

## Case presentation

A 25-year-old African-American male with a medical history of asthma and polysubstance abuse presented to the emergency room with worsening dyspnea with exertion and bilateral lower extremity edema over the last month. He had injected heroin intravenously for years but stopped in the last week. He also reported a constant burning chest pain that was relieved by lying on his side and sitting upright.

In the emergency department, the patient was hypertensive with a blood pressure of 182/146 mmHg and tachycardic with a heart rate of 120 beats per minute. Blood urea nitrogen was 116 mg/dL and serum creatinine was 9.15 mg/dL. Laboratory analyses also revealed a serum albumin level of 2.2 g/dL, brain natriuretic peptide level of over 5,000 pg/mL, and hemoglobin of 6.5g/dL with mean corpuscular volume of 65.8 fL. A chest X-ray revealed that the patient was in hypertensive emergency with flash pulmonary edema and cardiomegaly. He was then started on a nicardipine intravenous infusion to control his blood pressure in light of hypertensive emergency.

Workup for suspected acute renal failure was completed and was remarkable for an erythrocyte sedimentation rate of 37 mm/hour. Kidney ultrasound showed echogenic kidneys consistent with kidney disease. Computed tomography (CT) of the chest, abdomen, and pelvis showed moderate abdominal and pelvic ascites, bilateral pleural effusions, and anasarca. Urinalysis was remarkable for spot protein over 500 mg/dL. The 24hour urine protein was collected, which demonstrated over 3,500 mg of protein. The patient was negative for HIV, and a panel of respiratory viruses, including SARS-CoV-2, was also negative. An acute hepatitis panel was ordered, which demonstrated positive hepatitis C antibody. Quantitative HCV analysis by nucleic acid amplification testing revealed 546,371 copies of RNA. HCV genotyping revealed type 1a or 1b.

**Figure 1:**
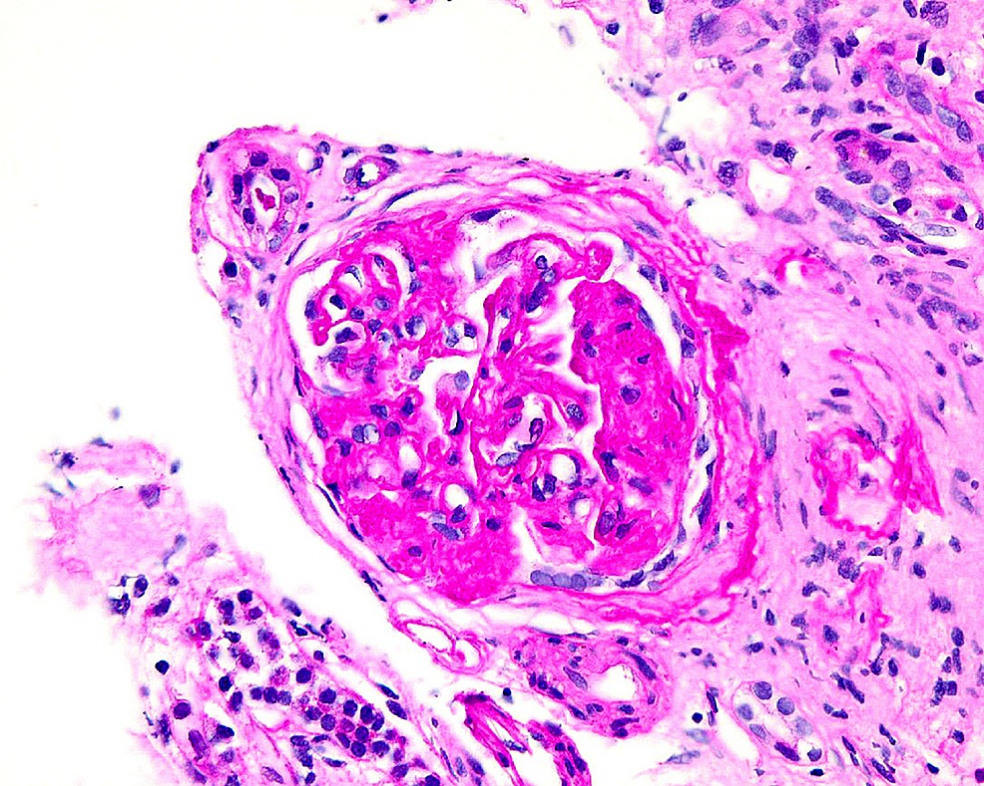
Light microscopy of renal biopsy demonstrating focal segmental glomerulosclerosis collapsing variant

**Figure 2:**
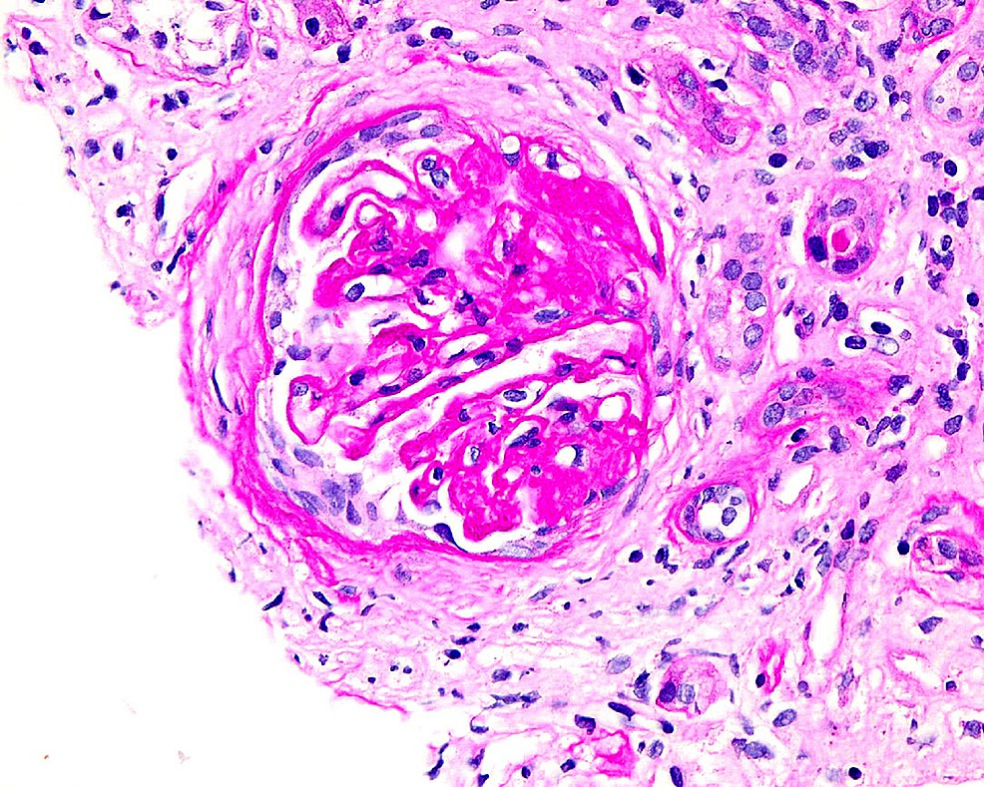
Light microscopy of renal biopsy demonstrating focal segmental glomerulosclerosis collapsing variant

Echocardiogram on admission showed an ejection fraction of 10% to 15%, grade III diastolic dysfunction, dilated right ventricle with decreased function, pulmonary hypertension, mild right and left atrial dilation, and pleural effusion. The patient had a Quinton catheter placed and received multiple sessions of hemodialysis. He eventually underwent a kidney biopsy that was significant for CG (Figures 1, 2). Prior to discharge, the patient had a tunnel hemodialysis catheter placed for outpatient hemodialysis. The patient was discharged with a LifeVest defibrillator and was given instruction to follow up with gastroenterology in the outpatient setting for HCV treatment.

## Discussion

CG is often described in association with HIV infection [12,13]; however, few case reports have shown it to be associated with HCV, although the incidence is unknown. It is worth noting that there have been multiple reports of de novo collapsing focal sclerosis in patients receiving treatment for HCV, including interferon therapy [5].

CG is also rarely seen in cases from other infectious sources, such as parvovirus, Epstein-Barr virus, cytomegalovirus, and SARS-CoV-2. There is potential for confounding factors in these cases, and further studies are required to investigate the pathophysiology of the role of HCV in CG.

FSGS is associated with segmental or global collapse of glomerular tufts. In most cases, there is sclerosis and collapse of the entire glomerular tuft [14]. Compared with a classical FSGS kidney biopsy, collapsing FSGS often shows marked hypertrophy and hyperplasia of the overlying glomerular epithelial cells, severe tubular injury, wrinkling, and retraction of the basement membrane [10,14].

The mechanisms leading to the collapsing pattern of glomerular capillaries, hypertrophic podocytes, and cystic dilation of tubules are not well understood.

Diagnosis of non-HIV collapsing FSGS should be suspected in patients presenting with nephrotic syndrome, especially if acute kidney injury is also present. A kidney biopsy is required to confirm the diagnosis and exclude other possible causes of the nephrotic syndrome. All patients should be HIV-negative by definition.

Treatment of CG variant of FSGS should focus on the underlying cause and supportive measures. In the setting of HCV-related FSGS, patients should be treated with antivirals. Patients typically will require lifelong dialysis, with compliance being significantly important. Angiotensin-converting enzyme inhibitors or angiotensin II receptor blockers should be prescribed to prevent further proteinuria. Statin therapy should be prescribed because of inherent risks of hyperlipidemia in the nephrotic syndrome. Diuretics such as furosemide along with a low sodium diet should be used to prevent edema and cardiorenal syndrome. Lastly, anticoagulation is usually required, as patients with nephrotic syndrome are at a high risk of venous thromboembolism.

Our findings indicate that HCV should be added to the differential diagnosis of patients with nephrotic syndrome, especially if FSGS is identified on biopsy. If a rare variant, such as CG is also identified, HCV should be considered as a possible inciting factor, as well as patients expressing the *APOL1* gene.

## Conclusions

Although HCV is rarely related to CG, it should be considered in patients diagnosed with the unique FSGS subtype of CG in light of findings of the aforementioned case. The present case was unique in that CG is a rare variant of FSGS and is sporadically associated with HCV in the literature and usually is associated with HIV infection. In this case, we highlight the utility in performing a kidney biopsy in the setting of nephrotic syndrome and acquiring a full renal workup. A full renal workup should include a viral hepatitis panel, as described in this case, because it was beneficial in finding a rare etiology for FSGS especially in the setting of the CG variant. Future studies including patients with acute renal failure in association with HCV are required to delineate the prevalence and incidence of HCV in CG variant of FSGS and to study how HCV-specific treatment affects underlying kidney disease and alters patient prognosis.
